# Altered Differentiation and Inflammation Profiles Contribute to Enhanced Innate Responses in Severe COPD Epithelium to Rhinovirus Infection

**DOI:** 10.3389/fmed.2022.741989

**Published:** 2022-02-25

**Authors:** Hong Guo-Parke, Dermot Linden, Aurelie Mousnier, Ian C. Scott, Helen Killick, Lee A. Borthwick, Andrew J. Fisher, Sinéad Weldon, Clifford C. Taggart, Joseph C. Kidney

**Affiliations:** ^1^Airway Innate Immunity Research Group, Wellcome Wolfson Institute for Experimental Medicine, School of Medicine, Dentistry and Biomedical Sciences, Queens University Belfast, Belfast, United Kingdom; ^2^Wellcome Wolfson Institute for Experimental Medicine, School of Medicine, Dentistry and Biomedical Sciences, Queens University Belfast, Belfast, United Kingdom; ^3^Translational Sciences and Experimental Medicine, Research and Early Development, Respiratory and Immunology, BioPharmaceuticals R&D, AstraZeneca, Cambridge, United Kingdom; ^4^Newcastle Fibrosis Research Group, Biosciences Institute, Faculty of Medical Sciences, Newcastle University, Newcastle upon Tyne, United Kingdom; ^5^Institute of Transplantation, Newcastle upon Tyne Hospitals, NHS Foundation Trust, Newcastle upon Tyne, United Kingdom; ^6^Department of Respiratory Medicine, Mater Hospital, Belfast, United Kingdom

**Keywords:** COPD, bronchial epithelial cells, ALI culture, human rhinovirus, differentiation, host innate response

## Abstract

**Background:**

Respiratory viral infections are closely associated with COPD exacerbations, hospitalisations, and significant morbidity and mortality. The consequences of the persisting inflammation and differentiation status in virus associated severe disease is not fully understood. The aim of this study was to evaluate barrier function, cellular architecture, the inflammatory response in severe COPD bronchial epithelium to human rhinovirus (HRV) induced pathological changes and innate immune responses.

**Methods:**

Well-differentiated primary bronchial epithelial cells (WD-PBECs) derived from severe COPD patients and age-matched healthy controls were cultured in the air-liquid interface (ALI) model. The differentiation phenotype, epithelial barrier integrity, pathological response and cytokine secreting profile of these cultures before and after HRV infection were investigated.

**Results:**

WD-PBECs derived from severe COPD patients showed aberrant epithelium differentiation with a decreased proportion of ciliated cells but increased numbers of club cells and goblet cells compared with healthy controls. Tight junction integrity was compromised in both cultures following HRV infection, with heightened disruptions in COPD cultures. HRV induced increased epithelial cell sloughing, apoptosis and mucus hypersecretion in COPD cultures compared with healthy controls. A Th1/Th2 imbalance and a strong interferon and pro-inflammatory cytokine response was also observed in COPD cultures, characterized by increased levels of IFNγ, IFNβ, IP-10, IL-10 and decreased TSLP and IL-13 cytokine levels prior to HRV infection. Significantly enhanced basolateral secretion of eotaxin 3, IL-6, IL-8, GM-CSF were also observed in both mock and HRV infected COPD cultures compared with corresponding healthy controls. In response to HRV infection, all cultures displayed elevated levels of IFNλ1 (IL-29), IP-10 and TNFα compared with mock infected cultures. Interestingly, HRV infection dramatically reduced IFNλ levels in COPD cultures compared with healthy subjects.

**Conclusion:**

An altered differentiation phenotype and cytokine response as seen in severe COPD WD-PBECs may contribute to increased disease susceptibility and an enhanced inflammatory response to HRV infection.

## Introduction

COPD is a heterogeneous respiratory syndrome consisting of two main disease phenotypes, chronic obstructive bronchitis and pulmonary emphysema (1–3). Cigarette smoke or noxious gases/particulate are key risk factors in the development of COPD (2, 3). A chronic persistent pulmonary inflammation, which is further exaggerated by a protease/anti-protease imbalance and oxidative stress, are prominent characteristics of COPD pathogenesis (1, 4). Consequently, the sustained inflammatory response contributes to abnormal tissue repair, mucus hypersecretion, and epithelial cell hyperplasia. This causes irreversible damage and thickening of the small conducting airway, which leads to progressive airflow limitation (1–4).

As a global epidemic, COPD has become the third leading cause of death worldwide, imposing an enormous healthcare and economic burden (2). Severe COPD, often accentuated with episodes of acute exacerbations, contributes to recurrent hospitalisations, morbidity and mortality (5). More than half of these exacerbations are associated with a range of respiratory viral infections (5–7). The common cold virus, human rhinovirus (HRV), is the major trigger of COPD acute exacerbations, accounting for 60% of viral-induced COPD exacerbations (8, 9). Other factors precipitate the onset of exacerbations including bacterial infections or environmental factors (5, 8, 9).

Bronchial epithelial cells are the first line of host defense and play a pivotal role in maintaining lung homeostasis and orchestration of the innate and adaptive response against inhaled smoke insults (7, 10, 11). The airway epithelium generates an inflammatory response by secreting cytokines and chemokines and recruiting immune cells (7, 11). The bronchial epithelium consists of a number of distinct subtypes of cells including ciliated cells, secretory cells (goblet and club cells) and basal cells (10, 11). These contribute to host defense by the maintenance of barrier function and mucociliary clearance. They also produce an array of inflammatory mediators to modulate innate and adaptive immune responses, tissue repair and remodeling (10–13). In COPD, cigarette smoke induces extensive destruction of the small airway epithelial wall and disruption of barrier integrity, resulting in impaired cilia beating capacity, and reduced mucociliary clearance (7, 11).

Respiratory viral infections target airway epithelium. HRV is classified into three genetically distinct strains (A, B, and C) with approximately 160 serotypes. Around 90% of HRV A and B utilize ICAM-I as a receptor thus referred to as the “major” receptor group (14). In addition, LDLR is the receptor for HRV minor group and CDHR3 for HRV strain C. HRV16 belongs to the major group of rhinoviruses and uses ICAM-1 on the surface of airway epithelial cells to enter and invade host cells (14). Upon virus entry, healthy airway epithelium secretes a range of cytokines and chemokines including IL-8, IL-6, TNFα, IFNα, RANTES, GM-CSF, and IL-1 resulting in immune cell accumulation at the site of the inflammation (4, 7, 9–11). In COPD, these factors heighten the inflammatory burden in the small airways, overpowering host anti-inflammatory mechanisms. This leads to epithelial cell sloughing, microvascular dilatation, oedema and goblet cell hyperplasia, the latter via the NOTCH signaling pathway (4, 7, 11, 15). There is also increased susceptibility of the small airway to bacterial infection (7, 9, 11). A previous study has demonstrated increased secretion of cytokines such as IL-6, IL-8 and Groα by COPD PBECs compared to healthy control cultures (16). Type I IFNs (IFNα/β) and type III IFNs (IFNλ) are the predominant interferons (IFNs) secreted by airway epithelium with the capacity to induce potent antiviral responses through IFN-stimulated genes (17, 18). Impaired type I and type III IFN responses have been described in bronchial epithelial cells in patients with asthma and COPD, contributing to the increased susceptibility to severe disease after viral infection (17–19).

Despite increasing awareness of the link between viral infection-associated COPD exacerbation and disease severity (12, 13), the impact of host factors on pathological changes in severe COPD bronchial epithelium during viral infection are not completely understood, in particular, factors linking susceptibility to severe disease. We hypothesize that changes in cell phenotype and number, barrier dysfunction and an altered cytokine response in severe COPD bronchial epithelium may contribute to increased rhinovirus-induced innate immune responses that may result in increased susceptibility to severe disease.

To address this, we exploited a well-differentiated primary bronchial epithelial cell (WD-PBEC) air-liquid interface (ALI) model to compare the phenotype of cultures from severe COPD patients with those from age-matched healthy controls. Epithelial cell differentiation subtype profiles and tight junction integrity were compared. The impact of HRV infection on pathological changes in bronchial epithelial cells in terms of virus growth kinetics, tropism, and epithelial cell sloughing and cytokine and chemokine secretion, were investigated. A greater understanding of the cause of alterations in COPD WD-PBEC function could facilitate the development of novel therapeutics to reduce disease morbidity and mortality.

## Materials and Methods

### Sample Information and Ethics Statement

This study was approved by the Newcastle and North Tyneside Local Regional Ethics Committee (11/NE/0291) and informed written consent from all study patients. COPD PBECs were derived from lung tissue of severe COPD patients (*n* = 7) undergoing either double or single lung transplants at the Freeman Hospital, Newcastle upon Tyne. The clinical characteristics of COPD patients are detailed in Table 1. Age matched healthy control PBECs were purchased from Lonza or PromoCell.

**Table 1:** COPD patient characteristics.

**No**.	**Age**	**Sex**	**FEV_**1**_ (L)**	**FVC (L)**	**FEV_**1**_/FVC**	**TLCO**	**Pack years**
	**(yr)**		**(% predicted)**	**(% predicted)**		**(% predicted)**	
1	46	M	0.48 (13%)	2.26 (50%)	21.20%	40%	130
2	62	M	0.36 (12%)	2.40 (61%)	15%	32%	40
3	58	M	0.52 (14%)	2.14 (44%)	24.20%	34%	80
4	58	M	0.32 (10%)	2.30 (60%)	13.90%	55%	60
5	64	M	0.52 (16%)	2.29 (54%)	22.70%	15%	30
6	62	M	0.67 (18%)	2.59 (55%)	25.90%	25%	70
7	60	M	0.57 (18%)	2.73 (66%)	20.90%	44%	40

*All patients had their lungs removed at transplantation. Patient age is given in years (yr). The spirometry for each patient is recorded in L and % predicted. The TLCO is the CO diffusion capacity shown as % predicted. All had been smokers, but had stopped prior to being accepted for transplantation. Pack years refers to the smoking history. FEV1, forced expiratory volume in 1 sec; FVC, forced vital capacity; TLCO, transfer factor for carbon monoxide*.

### Well-Differentiated Bronchial Epithelial Cell Culture

The generation of WD-PBEC cultures has been described elsewhere (18, 19). Briefly, PBECs obtained from severe COPD patients undergoing lung transplantation or age matched healthy controls (Lonza or PromoCell) were expanded in collagen-coated flasks. Cell monolayers were cultured in airway epithelial cell growth medium (Cat # C-21160) and Supplement Pack (Cat # C-39160) (PromoCell GmbH, Heidelberg, Germany) with additional penicillin and streptomycin. When 80% confluence was reached, the cells were seeded onto collagen-coated semipermeable transwells (Corning, NY) (6 mm diameter, 0.4 μm pore size) at 5 x 10^4^ cells/transwell and cultured in modified PromoCell airway epithelial cell growth and air-liquid interface culture medium as previously described (20, 21). At 100% confluence, the apical medium was removed and the cells were maintained in ALI for a minimum of 28 days. The medium in the basolateral compartment of each transwell was changed on alternate days.

### Virus Inoculation and Titration

HeLa H1 (ATCC CRL-1958, American Tissue Culture Manassas, VA, USA) cell lines were maintained in high glucose DMEM (Gibco) supplemented with 10% FBS (v/v). Human rhinovirus RV-A16 (a kind gift from Dr. Aurelie Mousnier, Queen's University Belfast) was propagated in HeLa H1 cells as detailed elsewhere (22). Virus titrations of HRV stocks and experimental samples were performed using a tissue culture infectious dose 50 (TCID_50_) assay as described previously (20, 23).

WD-PBEC cultures were either mock-infected (media only) or infected with HRV16 in duplicate. A multiplicity of infection (MOI) of 1 was used in the cultures. In brief, the apical surfaces of WD-PBECs were washed twice with 200 μl DMEM low glucose (no additives) to remove surface mucus prior to HRV infection. Then 100 μl of HRV16 inoculum or media-only were added to the apical surface of WD-PBEC cultures and incubated for 6 h at 33°C, 5% CO_2_. Subsequently, the inoculum was removed and the apical surface was washed six times with 250 μl low glucose DMEM (Gibco) with no FBS added. After the final wash, the apical surface rinses of the 6th wash were harvested, snap frozen in liquid nitrogen and stored at −80°C. Simultaneously, the basal medium (300 μl) of each culture transwell was harvested, snap frozen and stored at −80°C. Harvested medium was replaced with 300 μl of fresh ALI medium (Promocell). Apical washes and basal medium were also collected at desired time points after 6 hpi following the same procedures except the apical compartment was only washed once. All cultures were returned to a 37°C incubator with 5% CO_2_ after HRV inoculation and thereafter. HRV growth kinetics were determined by virus titration in apical washes at 6, 12, 24, 36, 48, 60, and 72 hpi. Basal medium was used to determine cytokine/chemokine responses. All cultures were monitored daily by light microscopy using Nikon Eclipse T5100 or TE-2000 U (Nikon, UK).

### Measurement of Trans-epithelial Electrical Resistance

On day 28 of ALI culture, as well as before and after HRV infection, Trans-epithelial Electrical Resistance (TEER) was measured using an EVOM2 and ENDOHM 6 mm chamber (World Precision Instruments). Briefly, transwells were gently rinsed with DMEM (without FBS) prior to the measurement. Afterwards, 250 μl of DMEM was added apically into the insert and measurements were performed according to the manufacturer's instructions as detailed previously (24, 25). When the cultures were fully differentiated, transwells with robust differentiation features, including TEERs above 300 Ω^*^cm^2^, extensive coverage of beating cilia, and obvious mucus production evidenced under microscope, were selected for further experimentation.

### Cytospin and Apoptosis Assay

To determine epithelial cell sloughing due to cytopathic effect, cytospins of apical washes were performed as previously described (21, 24). Briefly, apical washes of 250 μl of DMEM (without FBS) were added to a cytofunnel (EZ single cytofunnel, ThermoFisher Scientific, Waltham, MA) and spun at 1,000 rpm (ThermoShandon Cytospin 4 Cytocentrifuge) for 3 min onto a microscope slide. Cytospin slides were subsequently fixed in 4% (v/v) PFA for 15 min at room temperature (RT). Fixed slides were stored in the dark at −20°C until immunofluorescence was performed. Nuclei were stained using DAPI-mounting medium (Vectashield, Vector laboratories, USA). Apoptosis in sloughed cells was detected after cytospin with the terminal deoxynucleotidyl transferase dUTP nick end labeling (TUNEL) system, as per manufacturer's instructions (Roche, Hertfordshire, UK). Total DAPI^+^ and total TUNEL^+^ cells were counted under UV microscopy (Nikon TE-2000U and Hammamatsu Orca-ER camera).

### Phase Contrast and Immunofluorescence Analysis

Phase contrast images were obtained at 20x magnification of live COPD and healthy control cultures at 28 day post-ALI using a Nikon Eclipse TE-2000U microscope. Transwells were washed x3 with PBS for 5 min prior to permeabilisation in 0.2% (v/v) Triton X-100 (Sigma-Aldrich, Dorset, UK) in PBS (pH 7.4) for 30 min at RT. They were subsequently blocked with 2% (w/v) bovine serum albumin (BSA) (Sigma-Aldrich) in PBS (pH 7.4) for 1 h at RT. The wells were stained for airway epithelial cell differentiation subtype markers using rabbit anti-Muc5Ac antibody (ab78660, Abcam, Cambridge, UK), mouse anti-β-tubulin antibody (ab11309, Abcam), mouse anti-CC10 (sc-365992, Santa Cruz Technology, Dallas, TX), rabbit anti-ZO-1 antibody (Cat # 61-7300, Thermo Fisher Scientific, Waltham, MA), mouse anti-ICAM-1 (sc-8439, Santa Cruz Technology). In addition, HRV was detected using mouse anti-human rhinovirus VP2 (Cat # 18758, QED Bioscience, San Diego, CA).

All primary antibodies were diluted 1:100 in blocking buffer and incubated overnight at 4°C. The cells were washed x3 for 5 min in PBS before the addition of 100 μl anti-rabbit or anti-mouse secondary antibody (A11011, Alexa-Fluor 568, Invitrogen, Waltham, MA) at 1:500 dilution in 2% BSA (Sigma-Aldrich) in PBS at RT for 2 h. Cells were then washed x3 for 5 min in PBS prior to the addition of DAPI-mounting medium (Vectashield, Vector Laboratories, Burlingame, CA). Quantification of the percentage of ciliated, goblet, club cell numbers was carried out by counting under fluorescent microscopy (Leica DM5500). A minimum of 5 fields were captured per slide and the proportion of ciliated, goblet and club cells relative to total DAPI^+^ cell numbers was determined. Fluorescent images of well differentiated transwells staining for ciliated cells (anti-β-tubulin), goblet cells (anti-Muc5Ac), club cells (anti-CC10), HRV VP2, ZO-1, ICAM-1, were obtained using a Leica SP5 confocal DMI 6000 inverted confocal microscope (Leica Microsystems, Wetzlar, Germany).

### Cytokine and Chemokine Responses

Levels of eotaxin 3, GM-CSF, IL-6, IL-8, IL-10, IL-13, IP-10, IFNβ, IFNγ, TNFα and TSLP were quantified in basolateral media from WD-PBEC ALI cultures using custom multiplex immunoassays (Meso Scale Discovery, Rockville, MD, USA). All assays were undertaken according to the manufacturer's instructions. IL-29 (IFNλ1) concentration in basolateral medium was measured using human IL-29 ELISA (eBioscience, UK) according to manufacturer's instructions.

### Statistical Analysis

All data were analyzed using GraphPad Prism 8.0 (GraphPad Software Inc., San Diego, CA) and are reported as mean ± SD unless otherwise stated. Data were tested for normality by Shapiro-Wilk test and analyzed using Mann-Whitney U test or unpaired *t*-test for between-group comparisons where appropriate. Statistical significance was determined when *P* < 0.05.

## Results

### COPD Cultures Display Altered Differentiation and Impaired Barrier Integrity

**Figure 1:**
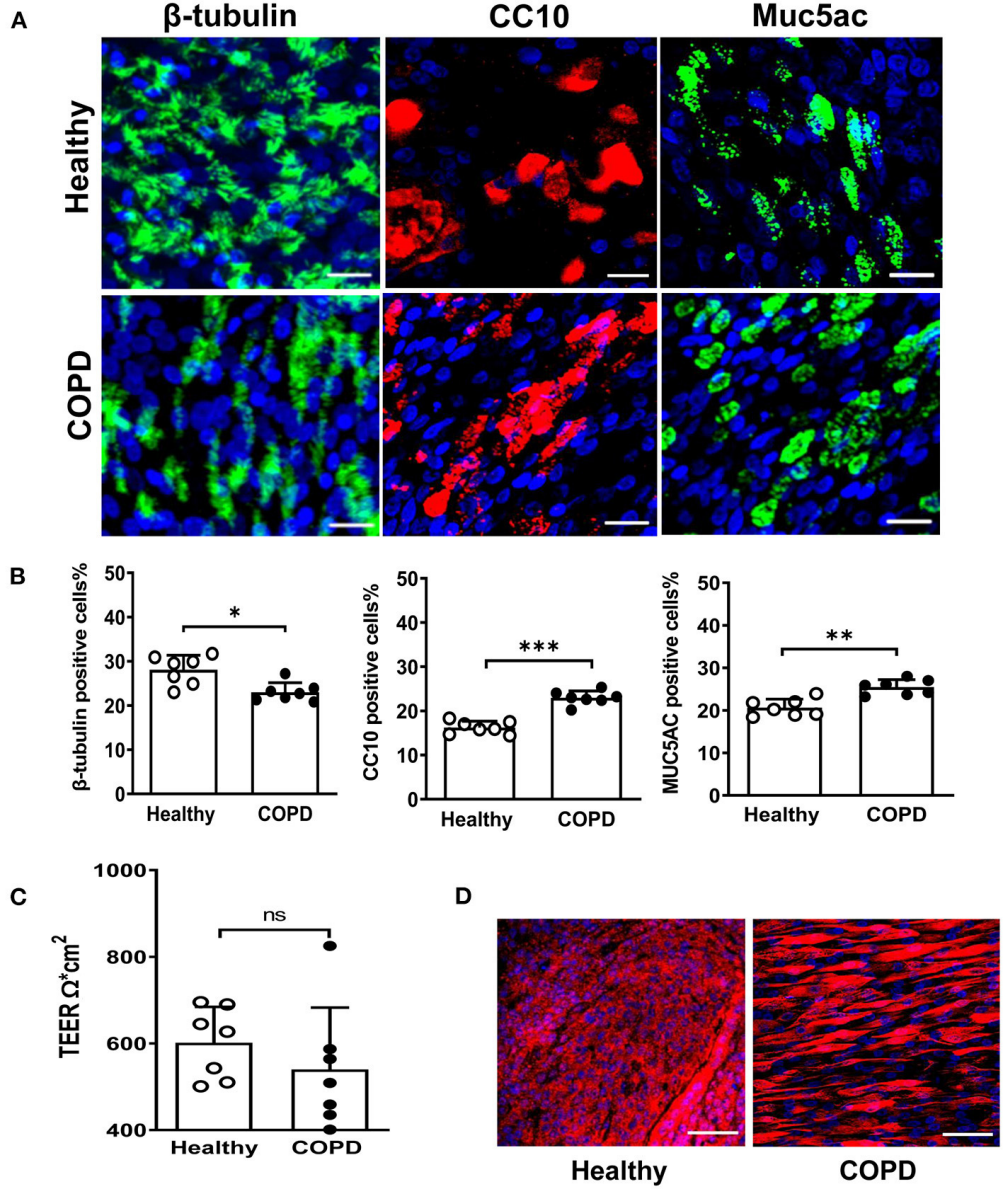
Altered differentiation and tight junction integrity in severe COPD cultures. **(A)** At 28 day post ALI, fully differentiated cultures derived from healthy donors and severe COPD patients were stained for ciliated cell marker β-tubulin (green), club cell marker CC10 (red), goblet cell marker Muc5Ac (green) and total DAPI^+^ cells (blue). Representative *en face* images of each protein were captured by SP5 confocal microscopy (magnification 63x with 1.46 digital zoom), scale bar 20 μm. **(B)** The abundance of each epithelial cell type was determined by counting the number of β-tubulin^+^ (ciliated) cells, CC10^+^ (club) cells, and Muc5Ac^+^ (goblet) cells in 5 random fields (in triplicate transwells) expressed as a percentage of the total number of cells counted [stained DAPI^+^ (blue)] per field (magnification 20x). Results are presented as mean ± SD, *n* = 7 per group, *P < 0.05, **P < 0.01 and ***P < 0.001, healthy vs. COPD. **(C)** Tight junction integrity of the cultures was monitored by transepithelial electrical resistance (TEER) with 5-6 transwells from each donor (*n* = 7). Data are plotted as mean ± SD. **(D)** Immunofluorescent staining of tight junction protein ZO-1 at 28 days post ALI, scale bar 50 μm. Images were captured by SP5 confocal microscopy (magnification 63x).

COPD (*n* = 7, age 58.6 ± 2.3 year) and healthy control (*n* = 7, age 61 ± 4.6 year) cultures were fully differentiated by day 28 post-ALI. Upon differentiation, they displayed indistinguishable typical cobblestone epithelium morphology and robust cilia coverage under light microscopy (Supplementary Figure S1). COPD WD-PBECs showed an altered cell differential profile compared to healthy WD-PBECs (Figures 1A,B). There was a reduction in the proportion of ciliated cells (β-tubulin) (23 ± 2.2 vs. 28.1 ± 3.3, *P* < 0.05), and an increase in club cells (CC10) (23 ± 1.6 vs. 16.2 ± 1.4, *P* < 0.001) and goblet cells (MUC5AC) (25.4 ± 1.8 vs. 20.6 ± 2.0, *P* < 0.01) in COPD cultures compared to healthy control cultures (Figure 1B). TEER values were lower in the COPD cells compared with healthy controls but did not reach significance (545 Ω^*^cm^2^ ± 145 vs. 602 Ω^*^cm^2^ ± 83, *P* = 0.20) (Figure 1C). More uniform ZO-1 staining was observed in healthy control cultures compared with COPD (Figure 1D).

### HRV16 Growth Kinetics, Tropism, and Impact on Barrier Integrity

**Figure 2:**
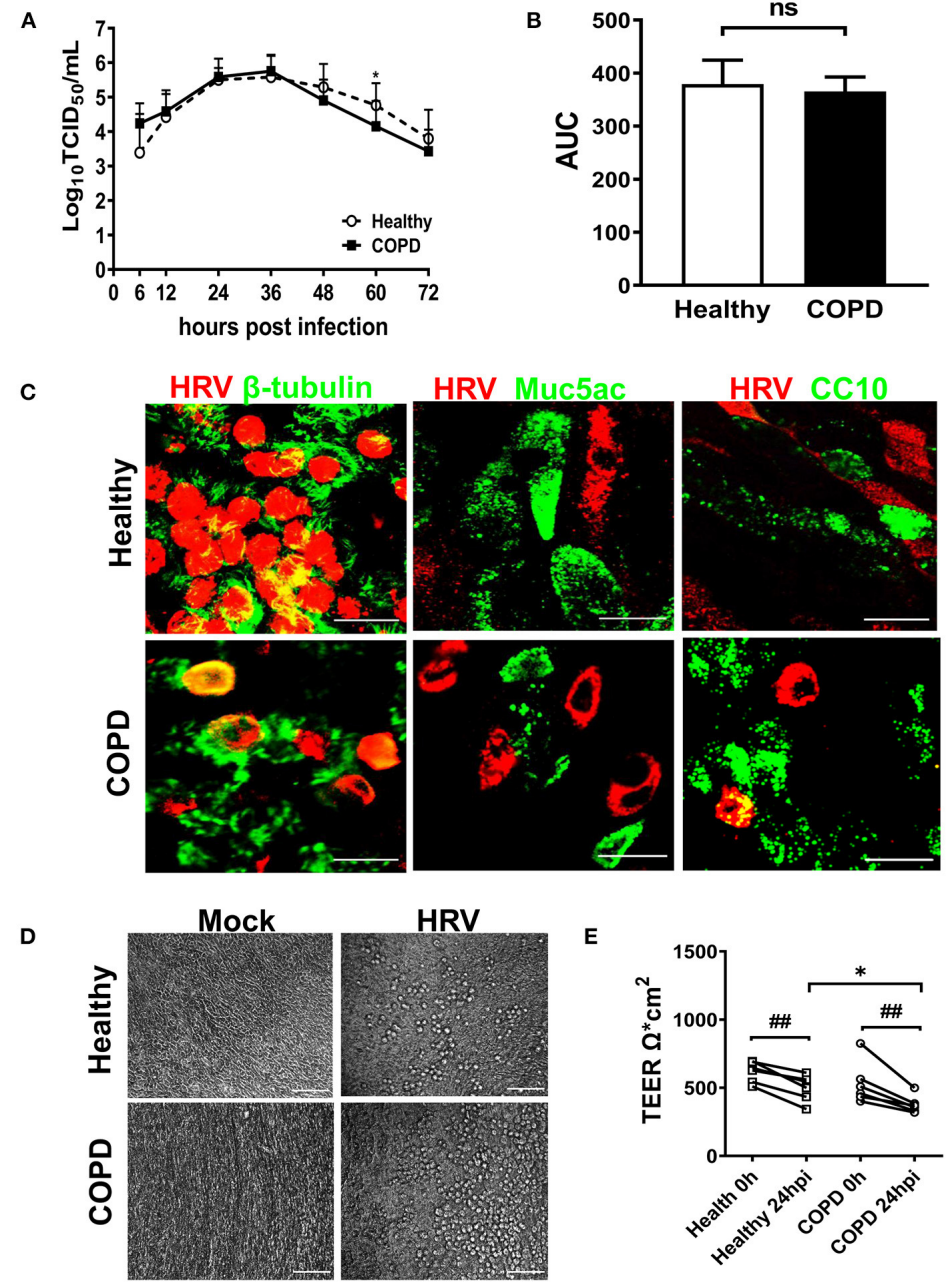
HRV16 growth kinetics, tropism and impact on cell integrity in cultures derived from healthy and severe COPD subjects. **(A)** Cultures from both groups (*n* = 6 per group) in duplicate were infected with HRV16 (MOI = 1) for 6 h at 33°C. At 6, 12, 24, 36, 48, 60, and 72 hpi, virus released from the apical compartments of the transwells were titrated by TCID50 assay for growth kinetics of HRV in WD-PBECs derived from healthy and severe COPD. Data are plotted as log10 mean ± SD. **(B)** HRV growth kinetics in healthy and COPD WD-PBECs were compared by calculating area under the curve (AUC). **(C)** To determine HRV tropism, cultured transwells in each group at 24 hpi (n = 6 in triplicate) were stained for β-tubulin (ciliated cells, green) and HRV VP2 protein (red), Muc5Ac (goblet cells, green) and HRV VP2 protein (red) or CC10 (club cells, green) and HRV VP2 protein (red). *En face* images were taken using SP5 confocal microscopy (magnification 63x with 3.0 digital zoom), scale bar 20 μm. **(D)** HRV-induced cytopathic effects were monitored under light microscope at each time point. Representative phase contrast images from each group in both mock infected and HRV infected transwells were captured at 24 hpi (magnification 20x), scale bar 200 μm. **(E)** The impact of HRV infection on tight junction integrity was examined at 24 hpi by TEER and presented as mean ± SD, *P < 0.01 healthy vs. COPD, ^##^*P* < 0.01 mock vs. HRV infection.

We next investigated HRV growth kinetics, tropism and its impact on tight junction integrity. HRV-induced shedding was observed only in the apical wash of the cultures with no detectable virus observed in basolateral medium (data not shown). Despite elevated ICAM-1 expression in COPD cultures (Supplementary Figure S2), a comparable HRV replication profile was displayed in both cultures. Viral load peaked between 24-36 hpi and declined from 48 hpi onwards. Virus shedding was significantly decreased at 60 hpi in COPD cultures compared with healthy cultures (Figure 2A, *P* < 0.05). Mean area under the curve (AUC) values for HRV growth kinetics of healthy and COPD cultures were 379 (95% CI: 351–408) and 365 (95% CI: 348–383), respectively, *P* = 0.37 (Figure 2B).

HRV infection occurred primarily in ciliated epithelial cells and occasionally club cells with no infection of goblet cells observed (Figure 2C). When examined under light microscope, HRV infection caused minimal cytopathic effect on the epithelial morphology of both cultures. Upon infection from 12 hpi onwards, small scattered foci of rounded cells appeared in both cultures (Figure 2D). HRV infection caused significant disruptions in tight junction integrity in both cultures at 24hpi compared with corresponding mock infected controls (both *P* < 0.01, Figure 2E). However, there was a significant reduction in TEER values in COPD compared with healthy cultures following HRV infection (Figure 2E). At 24 hpi, TEER values were 377 Ω^*^cm^2^ ± 65 in COPD and 498 Ω^*^cm^2^ ± 96 in healthy control cultures (*P* < 0.05).

### The Effects of HRV on Airway Epithelial Cell Sloughing and Apoptosis

**Figure 3:**
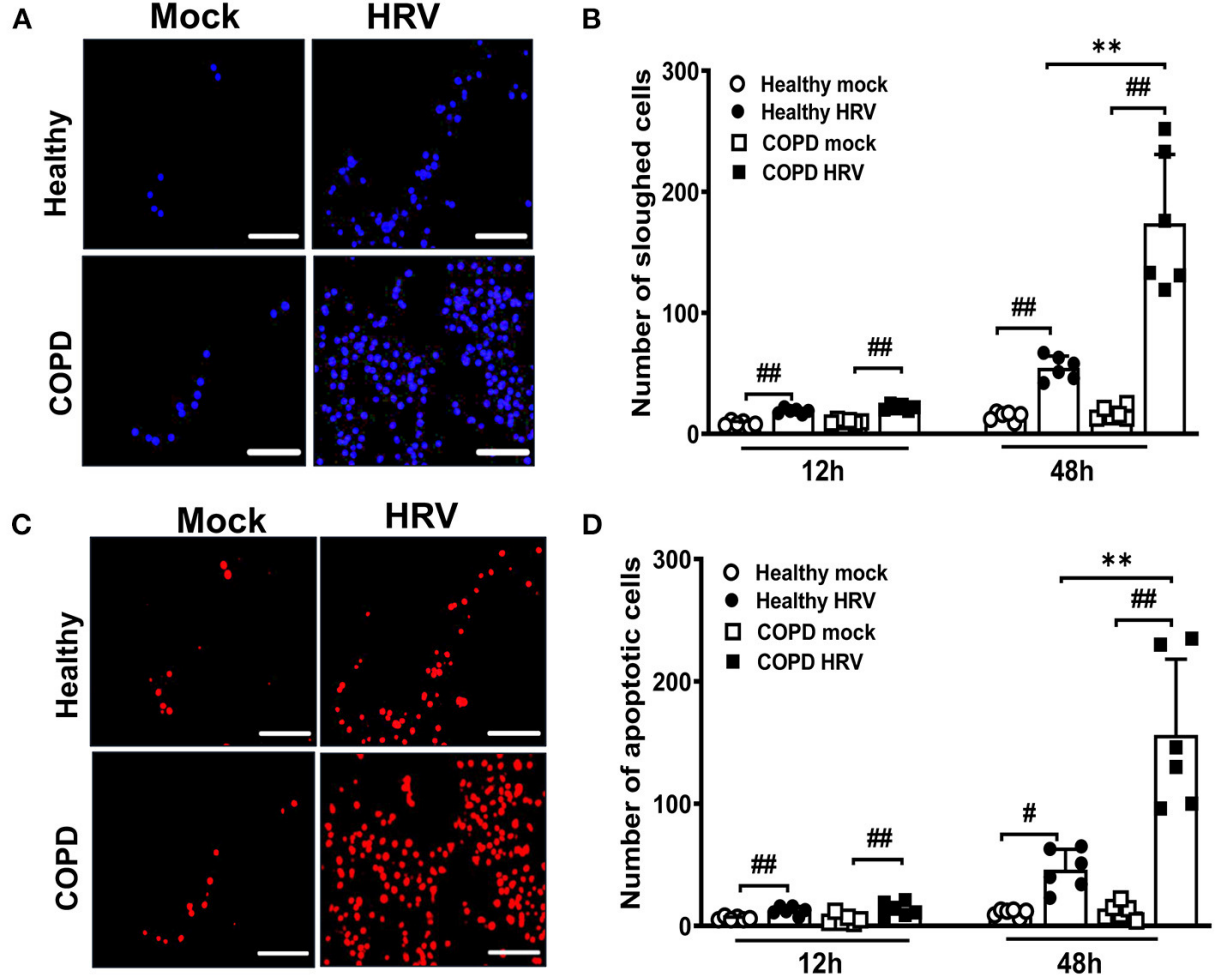
HRV infection augmented airway epithelial cells sloughing and apoptosis in severe COPD cultures. WD-PBEC cultures were mock-infected or infected with HRV (MOI = 1) and cytospins from apical washes were performed at indicated time points. **(A)** Cytospin slides were stained with DAPI at 48 hpi to visualize sloughed cells, representative images were recorded using Leica DM5500 (magnification 20x), scale bar 200 μm. **(B)** Quantification of the number of sloughed cells were determined by counting the number of DAPI^+^ nuclei in 5-10 random fields on each cytospin slide. Data are presented as mean ± SD, **P < 0.01 healthy vs. COPD, ^##^*P* < 0.01 mock vs. HRV infection. **(C)** Apoptosis in sloughed cells was detected using TUNEL assay, representative images were recorded using Leica DM5500 (magnification 20x), scale bar 200 μm. **(D)** Quantification of the number of apoptotic cells was performed by counting TUNEL^+^ cells in 5-10 random fields on each slide. Values are presented as mean ± SD, *n* = 6 per group, **P < 0.01 healthy vs. COPD. ^#^*P* < 0.05 and ^##^*P* < 0.01 mock vs. HRV infection.

Cell sloughing was increased after HRV infection of both cultures relative to corresponding mock-infected controls (Figures 3A,B). However, at 48 hpi HRV16 induced significantly more sloughing from COPD than healthy cultures (174 ± 57 vs. 55 ± 10, *P* < 0.01; Figure 3B). Furthermore, there was a greater increase in the number of TUNEL-positive apoptotic cells sloughed from both cultures after infection compared to corresponding mock-infected cultures, indicating HRV induced apoptosis (Figures 3C,D). Consistent with the relative numbers of sloughed cells, a significantly greater number of TUNEL-positive apoptotic cells were present in HRV-infected COPD cultures compared with healthy controls (156 ± 62 vs. 46 ± 17, *P* < 0.01; Figure 3D)

### HRV Induced Mucus Hypersecretion and Goblet Cell Hyperplasia

**Figure 4:**
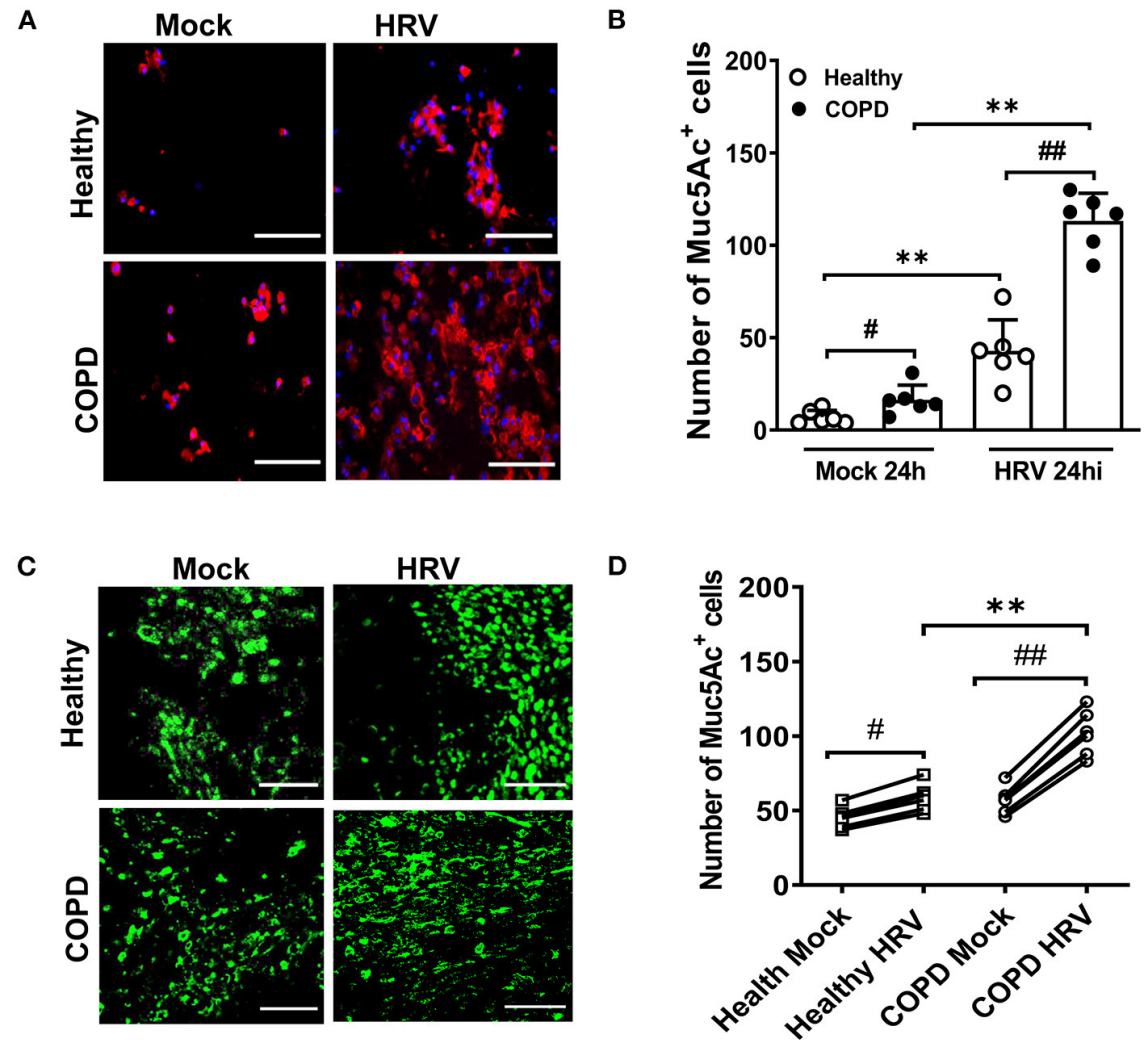
HRV infection induced mucus hypersecretion and goblet cell hyperplasia. **(A)** Cytospin slides at 24 hpi were stained for MUC5AC (red) to visualize mucus secretion. Representative images were recorded using Leica DM5500 (magnification 20x), scale bar 200 μm. **(B)** Quantification of Muc5Ac^+^ positive cells was performed for each condition at 24 hpi. Muc5Ac^+^ cells were counted in 10 different fields on each slide. Values are presented as mean ± SD, n = 6 per group, **P < 0.01 healthy vs. COPD, ^#^*P* < 0.05 and ^##^*P* < 0.01 mock vs. HRV infection. Transwells were **(C)** stained for MUC5AC (green) at 24 hpi (magnification 40x), scale bar 100 μm to determine goblet cell hyperplasia. **(D)** Quantification of Muc5Ac^+^ positive cells was performed for each condition at 24 hpi. Values are means ± SD, n = 6 per group, **P < 0.01 healthy vs. COPD, ^#^*P* < 0.05 and ^##^*P* < 0.01 mock vs. HRV infection.

Upon HRV infection, enhanced Muc5Ac was detected in sloughed cells of both cultures compared with corresponding mock infected cultures (7 ± 4 vs. 43 ± 17 and 16 ± 8 vs. 113 ± 15 for healthy and COPD cultures, respectively, both *P* < 0.01; Figures 4A,B). HRV increased globe cell sloughing in COPD cultures compared those in healthy culture (113 ± 15 vs. 43 ± 17, *P* < 0.01). At 24 hpi, goblet cell (Muc5ac staining cells) hyperplasia was observed in both HRV-infected healthy (59 ± 9 vs. 45 ± 7, *P* < 0.05) and COPD WD-PBECs (102 ± 15 vs. 57 ± 9, *P* < 0.01) compared to mock-infected cultures in the (Figures 4C,D). HRV exacerbated goblet cell hyperplasia in COPD cultures compared with healthy cultures (102 ± 15 vs. 59 ± 9, *P* < 0.01, Figures 4C,D).

### Severe COPD WD-PBECs Displayed Dysregulated Th1/Th2 Cytokine Secretion and Interferon Responses at Baseline and Following HRV Infection

**Figure 5:**
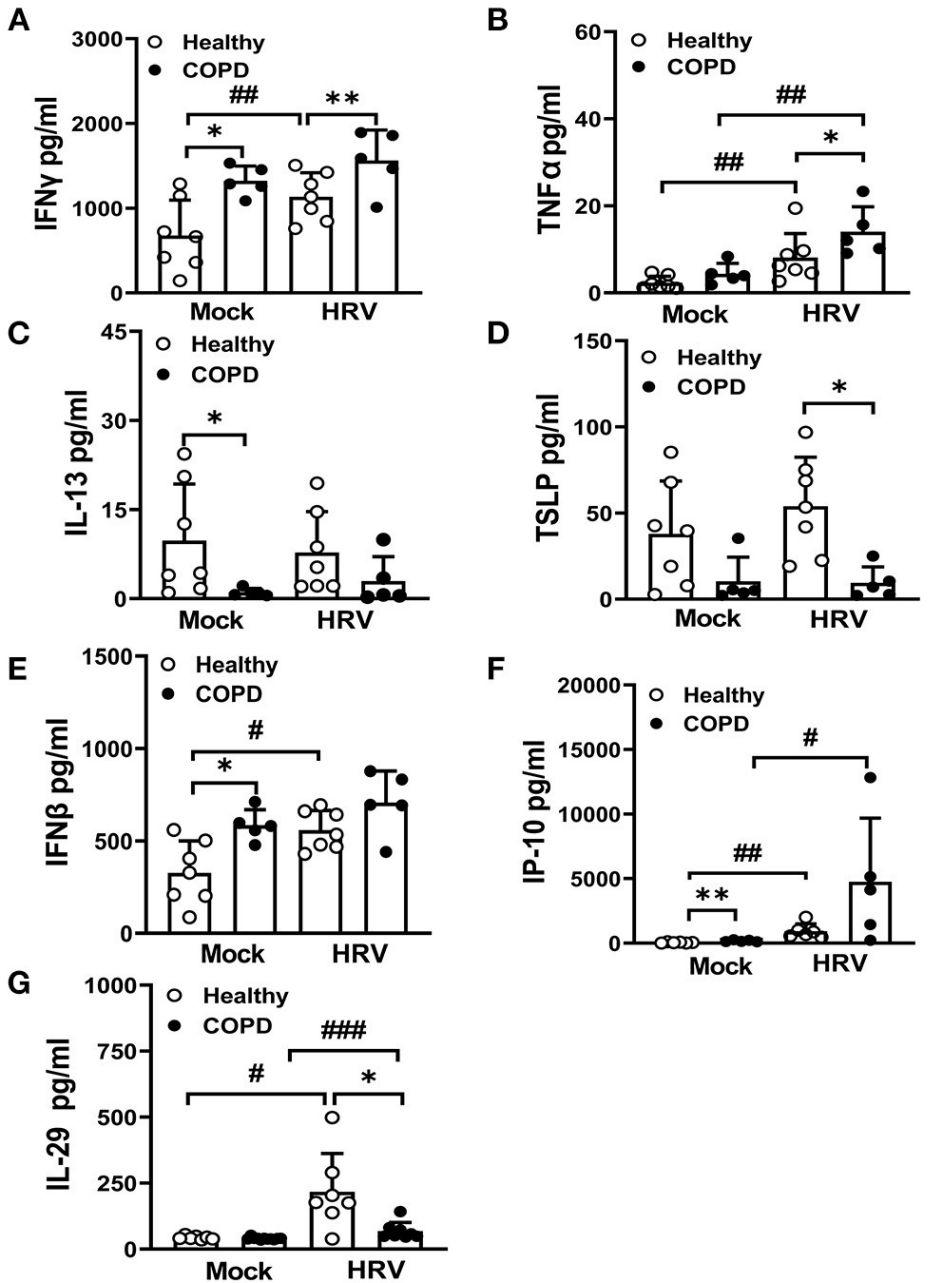
Dysregulated Th1/Th2 cytokine secretion and interferon responses in COPD cultures at baseline and following infection with HRV. At 24 hpi, basolateral medium of HRV- and mock-infected cultures was harvested and levels of Th1 cytokines, **(A)** IFNγ, **(B)** TNFα and Th2 cytokines, **(C)** IL-13, **(D)** TSLP quantified. Markers of interferon responses **(E)** IFNβ, **(F)** IP-10, **(G)** IL-29 were also analyzed. Values are mean ± SD, *n* = 7 for healthy WD-PBECs and *n* = 5 for cultures derived from severe COPD patients. *P < 0.05 and **P < 0.01 healthy vs. COPD, ^#^*P* < 0.05, ^##^*P* < 0.01 and ^###^*P* < 0.001 mock vs. HRV infection.

Baseline levels of the key Th1 cytokine, IFNγ, were significantly elevated in COPD basolateral secretions compared with healthy cultures (*P* < 0.05, Figure 5A). Upon infection, HRV further exaggerated the secretion of IFNγ (*P* < 0.01), and enhanced TNFα (another Th1 cytokine) production (*P* < 0.01) in COPD cultures compared to HRV infected healthy cultures (Figure 5B). Baseline secretion of IL-13, a key Th2 cytokine, was significantly reduced in mock infected COPD cultures compared to healthy cultures (*P* < 0.05, Figure 5C). In addition, levels of another Th2 cytokine, TSLP, were decreased (*P* < 0.05) following HRV infection of COPD cultures compared to those from healthy controls (Figure 5D).

Levels of epithelial-derived type I and III IFNs and interferon stimulated proteins were evaluated before and after infection. COPD cultures exhibited an increase in baseline levels of IFNβ (*P* < 0.05, Figure 5E) and IP-10 (*P* < 0.01, Figure 5F) compared with healthy cultures. However, HRV infection did not further enhance the levels of IFNβ in the COPD cultures, in contrast to IP-10 (Figures 5E,F). Although HRV infection significantly upregulated production of IFNλ responses, the HRV-induced IL-29 response was significantly lower in COPD cultures (*P* < 0.05) (Figure 5G).

### Elevated Levels of Cytokines/Chemokines Associated With COPD Pathogenesis

**Figure 6:**
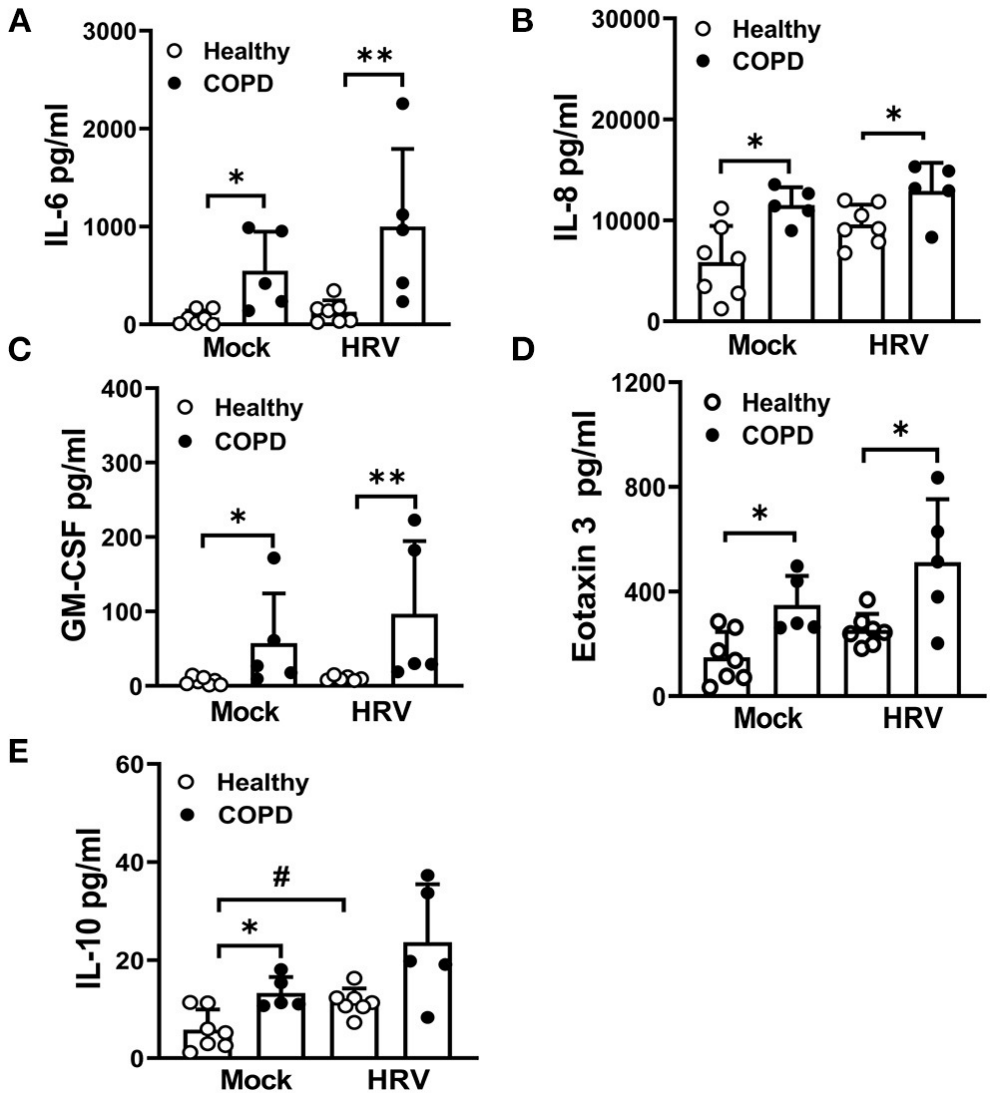
HRV exacerbates the secretion of cytokines/chemokines associated with COPD pathogenesis. At 24 hpi, levels of **(A)** IL-6, **(B)** IL-8, **(C)** GM-CSF, **(D)** eotaxin 3 and **(E)** IL-10 in the basolateral medium of HRV- and mock-infected cultures were measured. Values are mean ± SD, *n* = 7 for healthy WD-PBECs and n = 5 for cultures derived from severe COPD patients. *P < 0.05 and **P < 0.01 healthy vs. COPD, ^#^*P* < 0.05 mock vs. HRV infection.

Mock-infected COPD cultures displayed significantly elevated levels of IL-6, IL-8, GM-CSF, eotaxin 3 and IL-10, compared to mock infected healthy cultures (Figure 6). In response to HRV infection, levels of these mediators were marginally increased in COPD cultures compared with HRV infected healthy controls with the exception of IL-10, which increased in healthy (*P* < 0.05) but not COPD cultures following HRV infection (Figure 6E). In response to HRV infection, levels of RANTES were significantly increased in healthy cultures (*P* < 0.05) and a similar trend was observed in COPD cultures but did not reach significance (Supplementary Figure S3). A similar, albeit non-significant, effect was observed for IL-1α levels in HRV infected COPD cultures, whereas levels of IL-16 appeared to decrease (Supplementary Figure S3).

## Discussion

In the present study, we investigated the impact of the innate host response and barrier function of severe COPD WD-PBECs on HRV-induced innate immune responses. We observed differences in the numbers of ciliated, goblet and club cells in fully differentiated severe COPD cultures compared to cultures derived from healthy subjects. The bronchial epithelium is at the front line of host defense against respiratory virus-induced acute exacerbations in COPD and plays an important role in maintaining barrier integrity and the homeostasis of normal airway function (11, 17). Significantly reduced TEER values have been observed in cigarette smoke extract treated WD-PBEC cultures as well as fully differentiated cultures derived from COPD patients (12, 26). Under light microscopy, WD-PBEC in COPD cultures displayed very similar morphology, growth characteristics and multi-layered pseudostratified organization upon differentiation compared with healthy control cultures. However, differences in major cell subtype numbers were demonstrated in COPD cultures in terms of lower number of ciliated cells and higher numbers of Muc5Ac and club cells. These data were consistent with previous studies in COPD or cigarette smoke extract treated WD-PBEC cultures, which demonstrated aberrant epithelium differentiation as a result of decreased β-tubulin, increased Muc5Ac and increased CC10 expression (12, 26, 27). Rhinovirus is the most common contributor to upper and lower respiratory tract infection in adults and the predominant cause of virus induced acute exacerbations in COPD (6, 9, 16). Although HRV infection causes self-limiting, mild upper respiratory symptoms in a majority of infections, it also provokes acute bronchitis in severe cases. However, in COPD it can cause exacerbations with attendant risk of hospitalization and increased mortality (9, 16). We observed that HRV replication was restricted to ciliated cells. This agrees with recent reports that both HRV-A and HRV-C strains selectively infect ciliated cells in bronchial epithelial cells *in vitro* and *in vivo* (28, 29). Consistent with HRV infection in bronchial biopsies, a similar morphology, in terms of patchy infection, was observed in both healthy and COPD WD-PBEC cultures upon infection (29). Similar virus shedding has been reported in nasal washes in HRV-infected individuals with and without asthma (30). Despite higher levels of ICAM-1 in COPD at 24 hpi, similar levels of viral load were found in both culture cohorts. This may be explained by the differential constitution of the two cultures–although ICAM-1 levels are increased in COPD cultures this may be offset by decreased ciliated cells for virus tropism in COPD cultures. The decline in virus shedding observed in COPD cultures after peak replication suggests that initial viral clearance may occur following robust epithelial cell sloughing and apoptosis which in turn increases the severity of lung damage in COPD.

The functional abnormalities of COPD bronchial epithelium are associated with altered secretion of various inflammatory mediators, which may promote the infiltration of immune cells including neutrophils, macrophages and eosinophils thus orchestrating the chronic inflammatory response (6–9, 11). Elevated baseline levels of IFNγ, IFNβ, IP-10, IL-10 and decreased IL-13 were demonstrated in severe COPD cultures, indicating latent COPD inflammation may contribute to a Th1 cytokine-dominated inflammatory cytokine profile and elevated IFN-associated antiviral responses in airway epithelium. In addition, COPD cultures demonstrated up-regulated basolateral secretion of IL-6, IL-8, GM-CSF, and eotaxin 3, regardless of infection. It has been well established that high levels of IL-6 and IL-8 are strongly associated with the progression of airflow obstruction and the severity of emphysema (31, 32). Increased blood IL-6 is a common indicator of systemic inflammation of asthma and COPD, suggesting the therapeutic potential of IL-6 antagonists in COPD emphysema and associated complications (33–35). Elevated plasma IL-10 and eotaxin were also found in stable COPD subjects in a large scale cytokine and chemokine multiplex study (32). Increased GM-CSF, eotaxin, IP-10 have been documented in the sputum, blood, and lung tissue of COPD and correlated with disease severity and exacerbation frequency (36–38).

In contrast to the Th2 cytokine response demonstrated in some COPD studies (39, 40), our data shows a compromised Th2 response in terms of reduced baseline IL-13 secretion and an HRV-associated downregulation of TSLP in severe COPD cultures compared with healthy cultures. In contrast, IFNγ and TNFα levels were markedly increased in COPD cultures, indicating a Th1-dominated cytokine response in the severe COPD epithelium. Similarly, previous studies have also demonstrated a Th1 skewed cytokine response with enhanced airway IP-10 and its receptor CXCR3 (24, 41) and increased IFNγ and decreased IL-4 in the serum of COPD patients (42). Separately, IL-13 mRNA was shown to positively correlate with airway obstruction (FEV1 and FEV1/FVC) and impaired gas exchange and levels were lower in the lungs of patients with severe emphysema (43). Lower serum TSLP has also been demonstrated during acute COPD exacerbations compared with healthy controls (44).

Resolution of viral infection in the lung relies on the contribution of interferon responses that first occur at the site of infection. Consistent with a previous study in airway epithelial cells, impaired IFNλ1 (IL-29) expression was observed in COPD cultures upon HRV infection compared with healthy controls (19). In contrast to other studies demonstrating a deficiency of HRV-induced IFNβ production (17–19, 45, 46), our data demonstrates greater IFNβ secretion in mock and HRV-infected severe COPD cultures. However, HRV infection did not further reinforce this type I IFN response during HRV infection. As predicted, a rapidly increased IFNβ production was evident in healthy WD-PBECs upon HRV infection. The enhanced baseline levels of IFNβ observed in severe COPD cultures in our study could be explained by a pre-existing heightened production of IFNβ in severe COPD in response to persistent latent infection as described elsewhere (6, 7). Consistent with this findings, prolonged type I and type III IFN signaling has been implicated in disrupting lung epithelial integrity during recovery from viral infection (47).

It is worth noting that changes in respiratory epigenetic profiles such as DNA methylation, histone modification and microRNAs (miRNA) are likely to contribute to the altered differentiation and pro-inflammatory response in severe COPD. Perturbations in histone acetylation (HAT)/histone deacetylase (HDAC), methyltransferase (HMT) and histone demethylase (HDM) activities have been demonstrated to regulate many chromatin-dependent processes, including transcription, recombination and DNA repair (48–50). In COPD, cigarette smoke, accelerated aging and other environmental factors such as diet-modulated oxidative stress play a role in the pathogenesis of severe COPD (48, 49). Consequently, oxidativeg genome-wide epigenetic profiles in severe COPD airway epithelium would provide a great platform for potential the stress can regulate the activity of HATs and HDACs and enhance NF-B-dependent pro-inflammatory gene transcription such as IL-8 and TNFα (48–50). MiRNA-125a and –b have been shown to inhibit A20 and MAVS. to promote inflammation and impair antiviral response in COPD WD-PBECs (51). Understandinrapeutic anti-inflammatory targets in COPD and may help explain some of the findings in this study.

Taken together, our data suggests that severe COPD WD-PBECs display altered differential profiles, and elevated pro-inflammatory and IFN responses. HRV infection compromised the integrity of tight junction barrier, causing significant cell sloughing, epithelial apoptosis, mucus hypersecretion and heightened cytokine and chemokine secretory profiles in severe COPD cultures. Furthermore, there is an impaired type III IFN response upon HRV infection. The molecular changes in host epithelium and the pre-existing inflammatory environment, rather than viral replication *per se*, may contribute to the increased susceptibility of bronchial epithelium to respiratory virus-induced exacerbation in severe COPD. Targeting COPD inflammation and defects in host differentiation profiles may provide potential therapeutic targets in the prevention and management of severe COPD exacerbations.

## Data Availability Statement

The raw data supporting the conclusions of this article will be made available by the authors, without undue reservation.

## Ethics Statement

The studies involving human participants were reviewed and approved by Newcastle and North Tyneside Local Regional Ethics Committee (11/NE/0291). The patients/participants provided their written informed consent to participate in this study.

## Author Contributions

CT, JK, SW, AM, and HG-P designed the experiments and discussed and interpreted the results. HG-P, DL, LB, and HK performed experiments. HG-P analyzed data and wrote the manuscript. IS, LB, AF, CT, JK, and SW critically revised the draft. All authors have approved the final version of the manuscript for publication.

## Funding

This work was funded by the Mater Hospital Young Philanthropist (YP) trustees (to JK), Pfizer UK and Chiesi Farmaceutici (to CT and JK). Pfizer UK and Chiesi Farmaceutici were not involved in the study design, collection, analysis, interpretation of data, the writing of this article or the decision to submit it for publication.

## Conflict of Interest

IS and HK are employed by AstraZeneca. The remaining authors declare that the research was conducted in the absence of any commercial or financial relationships that could be construed as a potential conflict of interest.

## Publisher's Note

All claims expressed in this article are solely those of the authors and do not necessarily represent those of their affiliated organizations, or those of the publisher, the editors and the reviewers. Any product that may be evaluated in this article, or claim that may be made by its manufacturer, is not guaranteed or endorsed by the publisher.

## References

[1] Barnes PJ. Cellular and molecular mechanisms of asthma and COPD. Clin Sci (Lond). (2017) 131:1541–58. 10.1042/CS20160487 28659395

[2] Barnes PJ. COPD 2020: new directions needed. Am J Physiol Lung Cell Mol Physiol. (2020) 319:L884–6. 10.1152/ajplung.00473.2020 33050739

[3] Mannino DM Buist AS. Global burden of COPD: risk factors, prevalence, and future trends. Lancet. (2007) 370:765–73. 10.1016/S0140-6736(07)61380-4 17765526

[4] Barnes PJ. Inflammatory mechanisms in patients with chronic obstructive pulmonary disease. J Allergy Clin Immunol. (2016) 138:16–27. 10.1016/j.jaci.2016.05.011 27373322

[5] Ritchie AI Wedzicha JA. Definition, causes, pathogenesis, and consequences of chronic obstructive pulmonary disease exacerbations. Clin Chest Med. (2020) 41:421–38. 10.1016/j.ccm.2020.06.007 32800196 PMC7423341

[6] McManus TE Marley AM Baxter N Christie SN O'Neill HJ Elborn JS. Respiratory viral infection in exacerbations of COPD. Respir Med. (2008) 102:1575–80. 10.1016/j.rmed.2008.06.006 18672353 PMC7125807

[7] Linden D Guo-Parke H Coyle PV Fairley D McAuley DF Taggart CC. Respiratory viral infection: a potential “missing link” in the pathogenesis of COPD. Eur Respir Rev. (2019) 28:180063. 10.1183/16000617.0063-2018 30872396 PMC9488189

[8] George SN Garcha DS Mackay AJ Patel AR Singh R Sapsford RJ. Human rhinovirus infection during naturally occurring COPD exacerbations. Eur Respir J. (2014) 44:87–96. 10.1183/09031936.00223113 24627537

[9] Wilkinson TMA Hurst JR Perera WR Wilks M Donaldson GC Wedzicha JA. Effect of interactions between lower airway bacterial and rhinoviral infection in exacerbations of COPD. Chest. (2006) 129:317–24. 10.1378/chest.129.2.317 16478847 PMC7094441

[10] Ritchie AI Jackson DJ Edwards MR Johnston SL. Airway Epithelial Orchestration of Innate Immune Function in Response to Virus Infection. A focus on asthma. Ann Am Thorac Soc. (2016)13:S55–63. 10.1513/AnnalsATS.201507-421MG 27027954

[11] Gao W Li L Wang Y Zhang S Adcock IM Barnes PJ. Bronchial epithelial cells: The key effector cells in the pathogenesis of chronic obstructive pulmonary disease? Respirology. (2015) 20:722–9. 10.1111/resp.12542 25868842

[12] Schamberger AC Staab-Weijnitz CA Mise-Racek N Eickelberg O. Cigarette smoke alters primary human bronchial epithelial cell differentiation at the air-liquid interface. Sci Rep. (2015) 5:8163. 10.1038/srep08163 25641363 PMC4313097

[13] Kuek LE Lee RJ. First contact: the role of respiratory cilia in host-pathogen interactions in the airways. Am J Physiol Lung Cell Mol Physiol. (2020) 319:L603–19. 10.1152/ajplung.00283.2020 32783615 PMC7516383

[14] Bochkov YA Gern JE. Rhinoviruses and their receptors: implications for allergic disease. Curr Allergy Asthma Rep. (2016) 16:30. 10.1007/s11882-016-0608-7 26960297 PMC4854667

[15] Jing Y Gimenes JA Mishra R Pham D Comstock AT Yu D. NOTCH3 contributes to rhinovirus-induced goblet cell hyperplasia in COPD airway epithelial cells. Thorax. (2019) 74:18–32. 10.1136/thoraxjnl-2017-210593 29991510

[16] Schneider D Ganesan S Comstock AT Meldrum CA Mahidhara R Goldsmith AM. Increased cytokine response of rhinovirus-infected airway epithelial cells in chronic obstructive pulmonary disease. Am J Respir Crit Care Med. (2010) 182:332–40. 10.1164/rccm.200911-1673OC 20395558 PMC2921598

[17] Contoli M Message SD Laza-Stanca V Edwards MR Wark PA Bartlett NW. Role of deficient type III interferon-lambda production in asthma exacerbations. Nat Med. (2006) 12:1023–6. 10.1038/nm1462 16906156

[18] Wark PA Johnston SL Bucchieri F Powell R Puddicombe S Laza-Stanca V. Asthmatic bronchial epithelial cells have a deficient innate immune response to infection with rhinovirus. J Exp Med. (2005) 201:937–47. 10.1084/jem.20041901 15781584 PMC2213100

[19] Singanayagam A Loo SL Calderazzo M Finney LJ Trujillo Torralbo MB. Antiviral immunity is impaired in COPD patients with frequent exacerbations. Am J Physiol Lung Cell Mol Physiol. (2019) 317:L893–903. 10.1152/ajplung.00253.2019 31513433 PMC6962603

[20] Broadbent L Villenave R Guo-Parke H Douglas I Shields MD Power UF. In vitro modeling of RSV infection and cytopathogenesis in well-differentiated human primary airway epithelial cells (WD-PAECs). Methods Mol Biol. (2016) 1442:119–39. 10.1007/978-1-4939-3687-8_9 27464691

[21] Guo-Parke H Canning P Douglas I Villenave R Heaney LG Coyle PV. Relative respiratory syncytial virus cytopathogenesis in upper and lower respiratory tract epithelium. Am J Respir Crit Care Med. (2013) 188:842–51. 10.1164/rccm.201304-0750OC 23952745

[22] Papi A Johnston SL. Rhinovirus infection induces expression of its own receptor intercellular adhesion molecule 1 (ICAM-1) via increased NF-kappaB-mediated transcription. J Biol Chem. (1999) 274:9707–20. 10.1074/jbc.274.14.9707 10092659

[23] Subauste MC Jacoby DB Richards SM Proud D. Infection of a human respiratory epithelial cell line with rhinovirus. Induction of cytokine release and modulation of susceptibility to infection by cytokine exposure. J Clin Invest. (1995) 96:549–57. 10.1172/JCI118067 7615827 PMC185229

[24] Villenave R O'Donoghue D Thavagnanam S Touzelet O Skibinski G Heaney LG. Differential cytopathogenesis of respiratory syncytial virus prototypic and clinical isolates in primary pediatric bronchial epithelial cells. Virol J. (2011) 8:43. 10.1186/1743-422X-8-43 21272337 PMC3039598

[25] Parker J Sarlang S Thavagnanam S Williamson G. O'donoghue D, Villenave R, et al. A 3-D well-differentiated model of pediatric bronchial epithelium demonstrates unstimulated morphological differences between asthmatic and nonasthmatic cells. Pediatr Res. (2010) 67:17–22. 10.1203/PDR.0b013e3181c0b200 19755931

[26] Haswell LE Hewitt K Thorne D Richter A Gaça MD. Cigarette smoke total particulate matter increases mucous secreting cell numbers in vitro: a potential model of goblet cell hyperplasia. Toxicol In Vitro. (2010) 24:981–7. 10.1016/j.tiv.2009.12.019 20060463

[27] Gohy S Carlier FM Fregimilicka C Detry B Lecocq M Ladjemi MZ. Altered generation of ciliated cells in chronic obstructive pulmonary disease. Sci Rep. (2019) 9:17963. 10.1038/s41598-019-54292-x 31784664 PMC6884487

[28] Warner SM Wiehler S Michi AN Proud D. Rhinovirus replication and innate immunity in highly differentiated human airway epithelial cells. Respir Res. (2019) 20:150. 10.1186/s12931-019-1120-0 31299975 PMC6626354

[29] Mosser AG Vrtis R Burchell L Lee WM Dick CR Weisshaar E. Quantitative and qualitative analysis of rhinovirus infection in bronchial tissues. Am J Respir Crit Care Med. (2005) 171:645–51. 10.1164/rccm.200407-970OC 15591468

[30] Kennedy JL Shaker M McMeen V Gern J Carper H Murphy D. Comparison of viral load in individuals with and without asthma during infections with rhinovirus. Am J Respir Crit Care Med. (2014) 189:532–9. 10.1164/rccm.201310-1767OC 24471509 PMC3977713

[31] Zhang J Bai C. The significance of serum interleukin-8 in acute exacerbations of chronic obstructive pulmonary disease. Tanaffos. (2018) 17:13–21. 30116274 PMC6087525

[32] Bradford E Jacobson S Varasteh J Comellas AP Woodruff P O'Neal W. The value of blood cytokines and chemokines in assessing COPD. Respir Res. (2017) 18:180. 10.1186/s12931-017-0662-2 29065892 PMC5655820

[33] Peters MC McGrath KW Hawkins GA Hastie AT Levy BD Israel E. National heart, lung, and blood institute severe asthma research program. Plasma interleukin-6 concentrations, metabolic dysfunction,and asthma severity: a cross-sectional analysis of two cohorts. Lancet Respir Med. (2016) 4:574–84. 10.1016/S2213-2600(16)30048-0 27283230 PMC5007068

[34] Wei J Xiong XF Lin YH Zheng BX Cheng DY. Association between serum interleukin-6 concentrations and chronic obstructive pulmonary disease: a systematic review and meta-analysis. PeerJ. (2015) 3:e1199. 10.7717/peerj.1199 26336642 PMC4556145

[35] Ruwanpura SM McLeod L Dousha LF Seow HJ Alhayyani S Tate MD. Therapeutic targeting of the IL-6 trans-signaling/mechanistic target of rapamycin complex 1 axis in pulmonary emphysema. Am J Respir Crit Care Med. (2016) 194:1494–505. 10.1164/rccm.201512-2368OC 27373892

[36] Saha S Doe C Mistry V Siddiqui S Parker D Sleeman M. Granulocyte-macrophage colony-stimulating factor expression in induced sputum and bronchial mucosa in asthma and COPD. Thorax. (2009) 64:671–6. 10.1136/thx.2008.108290 19213775 PMC2712140

[37] Saetta M Mariani M Panina-Bordignon P Turato G Buonsanti C Baraldo S. Increased expression of the chemokine receptor CXCR3 and its ligand CXCL10 in peripheral airways of smokers with chronic obstructive pulmonary disease. Am J Respir Crit Care Med. (2002) 165:1404–9. 10.1164/rccm.2107139 12016104

[38] Baines KJ Fu JJ McDonald VM Gibson PG. Airway gene expression of IL-1 pathway mediators predicts exacerbation risk in obstructive airway disease. Int J Chron Obstruct Pulmon Dis. (2017) 12:541–50. 10.2147/COPD.S119443 28223794 PMC5308595

[39] Xia J Zhao J Shang J Li M Zeng Z Zhao J. Increased IL-33 expression in chronic obstructive pulmonary disease. Am J Physiol Lung Cell Mol Physiol. (2015) 308:L619–27. 10.1152/ajplung.00305.2014 25595648

[40] Yamada H Hida N Masuko H Sakamoto T Hizawa N. Effects of lung function related genes and *TSLP* on COPD phenotypes. COPD. (2020) 17:59–64. 10.1080/15412555.2019.1708296 31910693

[41] Majori M Corradi M Caminati A Cacciani G Bertacco S Pesci A. Predominant TH1 cytokine pattern in peripheral blood from subjects with chronic obstructive pulmonary disease. J Allergy Clin Immunol. (1999) 103:458–62. 10.1016/s0091-6749(99)70471-9 10069880

[42] Tang Y Guan Y Liu Y Sun J Xu L Jiang Y. The role of the serum IL-33/sST2 axis and inflammatory cytokines in chronic obstructive pulmonary disease. J Interferon Cytokine Res. (2014) 34:162–8. 10.1089/jir.2013.0063 24102578

[43] Castier Y Leçon-Malas Leçon-Malas V Fournier M Durand G Aubier M Dehoux M. Decreased expression of interleukin 13 in human lung emphysema. Thorax. (2004) 59:850–4. 10.1136/thx.2004.025247 15454650 PMC1746852

[44] Li W. Zhou Y, Zhang T. Expressions of serum TSLP, SAA and CRP in COPD patients. J Chin Phys. (2018) 20:46–9. 10.3760/cma.j.issn.1008-1372.2018.01.012

[45] Wu W Zhang W Booth JL Hutchings DC Wang X White VL. Human primary airway epithelial cells isolated from active smokers have epigenetically impaired antiviral responses. Respir Res. (2016) 17:111. 10.1186/s12931-016-0428-2 27604339 PMC5013564

[46] García-Valero J Olloquequi J Montes JF Rodríguez E Martín-Satué M Texidó L. Deficient pulmonary IFN-β expression in COPD patients. PLoS ONE. (2019) 14:e0217803. 10.1371/journal.pone.0217803 31170225 PMC6553750

[47] Major J Crotta S Llorian M McCabe TM Gad HH Priestnall SL. Type I and III interferons disrupt lung epithelial repair during recovery from viral infection. Science. (2020) 369:712–7. 10.1126/science.abc2061 32527928 PMC7292500

[48] Schamberger AC Mise N Meiners S Eickelberg O. Epigenetic mechanisms in COPD: implications for pathogenesis and drug discovery. Expert Opin Drug Discov. (2014) 9:609–28. 10.1517/17460441.2014.913020 24850530

[49] Gozzi-Silva SC Teixeira FME Duarte AJDS Sato MN Oliveira LM. Immunomodulatory role of nutrients: how can pulmonary dysfunctions improve? Front Nutr. (2021) 8:674258. 10.3389/fnut.2021.674258 34557509 PMC8453008

[50] Fathinavid A Ghobadi MZ Najafi A Masoudi-Nejad A. Identification of common microRNA between COPD and non-small cell lung cancer through pathway enrichment analysis. BMC Genom Data. (2021) 22:41. 10.1186/s12863-021-00986-z 34635059 PMC8507163

[51] Hsu AC Dua K Starkey MR Haw TJ Nair PM Nichol K. MicroRNA-125a and -b inhibit A20 and MAVS. to promote inflammation and impair antiviral response in COPD. JCI Insight. (2017) 2:e90443. 10.1172/jci.insight.90443 28405612 PMC5374076

