# Supplementary material for: Altered Differentiation and Inflammation Profiles Contribute to Enhanced Innate Responses in Severe COPD Epithelium to Rhinovirus Infection
Source: Front Med (Lausanne). 2022 Feb 25;9:741989. doi: 10.3389/fmed.2022.741989 (PMC8916560; doi:10.3389/fmed.2022.741989)
Supplement: Supplementary file 1 [file Data_Sheet_1.PDF]

Supplementary Material

Supplemental Methods

RNA Extraction, cDNA Synthesis and qPCR

On day 28 of ALI culture, total RNA was extracted cells from transwells using a miRNeasy Mini Kit (Cat # 217004, Qiagen UK). cDNA synthesis was carried out using the high-capacity cDNA reverse transcription kit (ThermoFisher Scientific, UK) following the manufacturer's instructions. Quantitative PCR (qPCR) reagents and cDNA was transferred from a source plate to a 384-well PCR plate using the Echo 525 liquid handling system (Labcyte, USA). PCR reactions were performed using inventoried TaqMan® gene expression assays (Cat # 4369016, ThermoFisher Scientific) for ICAM-1 (Hs00164932_m1) and GAPDH (Hs02786624_g1). qPCR was performed using a LightCycler® 480 Real-Time PCR System (Roche, UK). Relative expression of target genes relative to GAPDH was calculated using the $2(-\Delta Ct)$ method.

Cytokine and Chemokine Responses

RANTES, IL-1α, and IL-16 levels in basolateral media from WD-PBEC ALI cultures were determined using custom multiplex immunoassays (Meso Scale Discovery, Rockville, MD, USA). All assays were undertaken according to the manufacturer's instructions.

Supplemental Figures

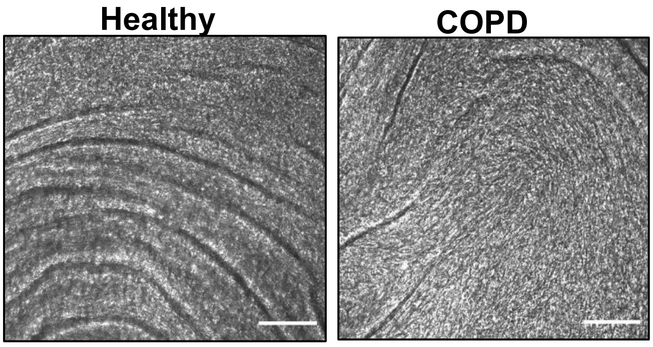

Figure S1. Fully differentiated cultures derived from COPD and healthy subjects displayed similar morphology. Representative images of transwell cultures from both groups were monitored at ALI day 28 by phase-contrast microscope (Nikon TE-2000U, original magnification x20), scale bar 200 µm.

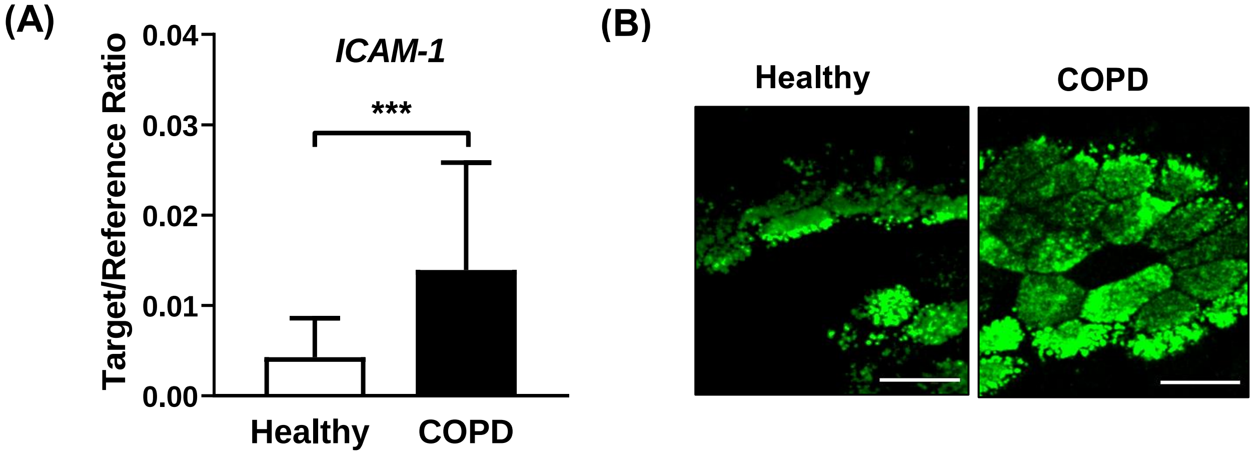

Figure S2. Upregulated ICAM-1 in severe COPD cultures. At 28 day post ALI culture, (A) transcriptional expression of HRV16 receptor ICAM-1 was performed by RT-qPCR (n = 7 in duplicate for each group). Data were plotted as mean ± SD, ***P<0.001. (B) ICAM-1 protein was detected by immunofluorescent staining. Representative *en face* images of each staining were captured under SP5 confocal microscopy (magnification 63x with 2.0 digital zoom), scale bar 20 µm.

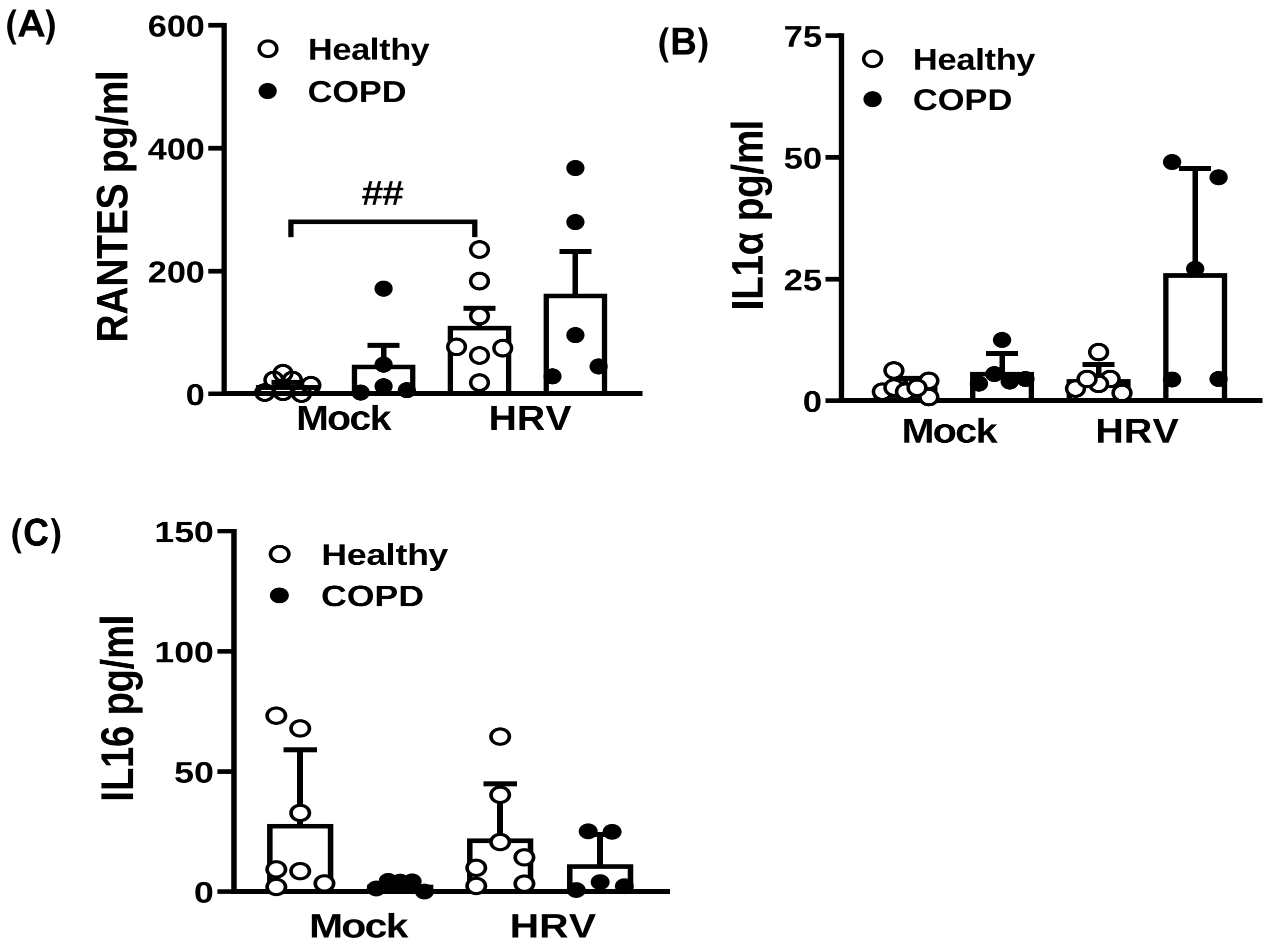

Figure S3. RANTES, IL-1α and IL-16 levels in response to HRV infection. At 24 hpi, the basolateral medium of HRV- and mock-infected cultures were harvested and levels of (A) RANTES (B) IL-1α and (C) IL-16 were quantified. Values are mean ± SD, n = 6 in duplicate for each condition. ##P<0.01.
